# Gas-y

**Published:** 1877-08

**Authors:** 


**GAS—Y.**—That fine lecturer, the missionary Fletcher, states that monkeys exhibited for a few days or nights in rooms lighted by gas, or warmed by coal-fires, will inevitably die in a very short time. This fact has been observed in Philadelphia ana at the Jardin des Plantes at Paris. Dr. Arnott reported of the Zoölogical Collection in London, a loss of fifty monkeys in about a month, from a like exposure, and an imperfect ventilation.

The ceilings of our dwellings are darkened, not so much by coal-fires or furnace-heat, as by the gas-lights; not necessarily, but from ignorance or inattention. Many persons, especially servants, in lighting a gas-burner, first turn the faucet or screw its full distance, and then apply the flame. Between the two acts, a considerable amount of unburnt gas escapes; and being light, it reaches the ceiling instantly in its carbonic or coaly state, and makes its dingy impress. Again, it is generally supposed that the more completely the gas is turned on, the more light will be given out. This is not so, except towards daylight, when the pressure on the gas-reservoirs is very much reduced. In the early part of the evening, the pressure is so great, that the gas escapes faster than it is burned, known by the rushing noise it makes. If the key is turned square, even if an indistinct singing is heard by putting the ear near the burner, it is gas escaping unburned. Such is the case, too, when the flame is spire-like, or jagged in sharp points. The more even the line of light is, the more perfectly is the gas consumed. Many persons who are too stupid to be reached by an appeal to the health, will jump with the utmost alacrity at the jingle of a dollar. Such are informed, then, that unburned gas not only kills men as well as monkeys, but it will inevitably darken and dinge their costly frescoes; and they had better turn the attention of their families and servants to the philosophical management of their gas-lights.

